# Retrospective real-world database study to examine the prevalence and incidence of cardiovascular diseases and medication prescription in Japanese patients with type 2 diabetes

**DOI:** 10.1007/s13340-026-00877-7

**Published:** 2026-03-17

**Authors:** Mitsuhisa Komatsu, Hiroshi Kobayashi, Satoshi Tsuboi, Hirotaka Watada

**Affiliations:** 1 Division of Diabetes, Endocrinology and Metabolism, Department of Internal Medicine, Shinshu University School of Medicine, Asahi 3–1-1, Matsumoto, Nagano 390–8621 Japan; 2 Cardiovascular and Emerging Therapy Areas, Medical Affairs Department, CMRQ Development Division, Novo Nordisk Pharma Ltd, Tokyo, Japan; 3 RWE Group, SPVD Department, CMRQ Development Division, Novo Nordisk Pharma Ltd, Tokyo, Japan; 4 Department of Metabolism and Endocrinology, Juntendo University Graduate School of Medicine, Tokyo, Japan

**Keywords:** Type 2 diabetes, Diabetes medications, GLP-1RA, SGLT-2i, 3P-MACE, Cardiovascular disease

## Abstract

**Introduction:**

In Japan, real-world characterisation of cardiovascular disease (CVD) prevalence, incidence, and medication prescription in adults with type 2 diabetes (T2D) has been limited.

**Methods:**

This real-world study (comprising a cross-sectional and a retrospective study) in adults aged ≥ 20 years with T2D used the Medical Data Vision database to evaluate the prevalence and incidence of three-point major adverse cardiovascular events (3P-MACE) and composite CVD and to describe medication prescription patterns.

**Results:**

The cross-sectional study included 292,383 patients in 2014 and 622,531 in 2021. Prevalence of 3P-MACE and composite CVD were 4.8% and 12.1%, respectively, in 2014; values in 2021 were 5.0% and 14.0%. In 2014, 2.1% of patients received glucagon-like peptide-1 receptor agonists (GLP-1RAs) and 0.8% received sodium-glucose cotransporter-2 inhibitors (SGLT-2is); 2021 values were 9.2% and 32.3%. The retrospective study included 314,206 patients; 19.7% had a CVD history. The overall incidence rate of 3P-MACE per 1000 person-years was 12.8 (95% confidence interval [CI] 12.6–13.0); 46.6 (45.3–48.0) in those with and 8.6 (8.4–8.8) in those without CVD history. The incidence rates (95% CI) of composite CVD were 47.9 (47.4–48.3); 179.5 (177.0–182.1) in those with and 31.5 (31.1–31.9) in those without CVD history.

**Conclusions:**

In Japan, the prevalence of 3P-MACE and composite CVD were almost unchanged from 2014 to 2021, whereas GLP-1RA/SGLT-2i prescriptions increased. Incidence rates of 3P-MACE and composite CVD were higher in patients with a CVD history. These data provide insights into prescription patterns in T2D in Japan and could inform future treatment practices.

**Supplementary Information:**

The online version contains supplementary material available at 10.1007/s13340-026-00877-7.

## Introduction

Type 2 diabetes (T2D) is a complex metabolic disorder characterised by hyperglycaemia and is associated with increased risk of cardiovascular disease (CVD), microvascular diseases, and other metabolic complications [1, 2]. Globally, 34.8% of adults with T2D have established CVD; in Japan, the prevalence of CVD in adults with T2D is 37.3% [3]. Although antidiabetes therapies with cardiovascular health benefits are available, the incidence of major adverse cardiovascular events (MACE) remains greater in people with T2D compared with those without T2D [4, 5]. Glucagon-like peptide-1 receptor agonists (GLP-1RAs) reduce circulating glucose levels by promoting insulin secretion and suppressing glucagon secretion from the pancreas [6], while also leading to augmented satiety through activation of neural pathways in the hypothalamus [7, 8]. Some GLP-1RAs have demonstrated beneficial effects in people with T2D and concomitant CVD and chronic kidney disease [9–13]. The mechanisms governing the cardiovascular benefits of GLP1-RAs are still being elucidated; however, in addition to benefits mediated through weight loss and glycaemic control, there is evidence that GLP-1RAs attenuate atherosclerotic lesions through anti-inflammatory mechanisms [14, 15].

Previous trials have demonstrated that GLP-1RAs such as liraglutide and semaglutide have cardiovascular benefits in patients with T2D and established CVD or high risk of cardiovascular events [11, 16, 17]. Sodium-glucose cotransporter-2 inhibitors (SGLT-2is) have been shown to have protective effects against CVD in people with T2D [18–20]. As such, both the American Diabetes Association and the Japan Diabetes Society recommend GLP-1RAs and SGLT-2is for individuals with T2D and concomitant CVD [21, 22]. However, globally, only 20% of people with T2D and established CVD are treated with a medication with proven cardiovascular benefits [3]. In Japan, between 2014 and 2017, GLP-1RAs and SGLT-2is were prescribed to 0.2% and 7.6% of people with T2D, respectively [23]. Monitoring changes in prescription patterns of these drug classes in Japanese adults with T2D and established CVD, and the corresponding real-world effect they have on CVD outcomes in these individuals could highlight changes in T2D care and improve outcomes and disease management.

Although emerging data from global clinical trials provide valuable insights into the use of different medications for the treatment of T2D with established CVD, there is a lack of data from a real-world setting. This is especially true in Japan where there has been limited characterisation of people with T2D and CVD. This study, comprising a cross-sectional study and a retrospective cohort study, aimed to describe demographic, treatment, and clinical characteristics, with a particular focus on those relating to CVD (three-point [3P]-MACE and a composite CVD outcome), in a real-world population with T2D in Japan.

## Methods

### Study design

This study comprised two component studies: a cross-sectional study and a retrospective cohort study. Both component studies used secondary data, provided by Medical Data Vision (MDV) Co., Ltd. (Tokyo, Japan) and collected from approximately 450 hospitals that had provided consent for secondary use of data. The MDV database includes information on disease diagnosis, lab results (available for approximately 10% of the study population), dosage and administration periods for medication prescriptions, and medical procedures.

The overall study period was 1 April 2008 to 30 June 2022. The cross-sectional study index date was recorded as the date of first prescription of antidiabetes medication within the same month as the first diagnostic record of T2D, per time period (1 January 2014–31 December 2014 and 1 January 2021–31 December 2021) (Fig. S1a). The index date for the retrospective cohort study was defined as the date of first prescription of antidiabetes medication within the same month as the diagnostic record of T2D (regardless of whether an inpatient or outpatient visit) on or after 1 April 2013 (Fig. S1b). Inclusion criteria in both component studies included an age of ≥ 20 years at index date and having ≥ 1 hospital visit) with a with diagnostic report of T2D using the International Classification of Disease 10th Revision (ICD-10; E11, E12, E13, E14); these criteria had to be met during each time period for the cross-sectional study, and on or after April 2013 for the retrospective cohort study. Both component studies had the exclusion criterion of a previous hospital visit(s) with diagnostic report of type 1 diabetes (ICD-10; E10); this was for prior to the index date for the retrospective study, and within the overall study period for the cross-sectional study. In the retrospective cohort study, an additional exclusion criterion was the lack of a look-back period of 12 months before the index date.

In both component studies, patient demographics (age, sex) and new diagnosis of T2D were reported at index date. Newly diagnosed T2D was defined as patients without a hospital visit with a diagnostic record of T2D prior to the index date (exclusive). Clinical characteristics (microvascular complications, hypertension, and hyperlipidaemia) and lab test data (glycated haemoglobin [HbA_1c_], estimated glomerular filtration rate [eGFR], and blood lipids) were also recorded in both component studies. The primary outcomes were prevalence of 3P-MACE and composite CVD outcome in the cross-sectional study, and incidence of 3P-MACE and composite CVD outcome in the retrospective cohort study. 3P-MACE was defined as a composite of cardiovascular death, non-fatal myocardial infarction (MI), and non-fatal stroke; composite CVD outcome was defined as the outcome of any of cardiovascular death, fatal/non-fatal MI, fatal/non-fatal stroke, coronary artery disease (CAD), heart failure (HF), peripheral artery disease (PAD), and atrial fibrillation (AF). Cardiovascular death included in-hospital death caused by MI, stroke, CAD, HF, PAD, or AF. Recorded CVD events were limited to inpatient cases to ensure accuracy. Healthcare resource utilisation (HCRU) was measured from the day after the index date to the end of the time period; it included both general and T2D/CVD-related number of outpatient visits, hospitalisations, and length of stay in hospital, as well as T2D/CVD-related lab tests.

In the cross-sectional study, patients were identified and examined in two 1-year time periods (1 January 2014–31 December 2014 and 1 January 2021–31 December 2021). Participant CVD status, medications (antidiabetes and cardiovascular-related), and clinical characteristics were measured. Prevalence of 3P-MACE and composite CVD outcome were measured over the entire time period regardless of index date and stratified by antidiabetes medication types. These time points were selected to assess any differences before and after the accumulation of evidence from cardiovascular outcome trials containing data related to glucose-lowering medication usage patterns. In the retrospective cohort study, antidiabetes medication prescriptions were measured ± 30 days of the index date. Cardiovascular-related medications and clinical characteristics were measured for the year prior to and including the index date, and 3P-MACE, composite CVD outcome, and HCRU were measured during follow-up.

### Statistical analysis

Both component studies were descriptive, and no formal hypotheses were tested therefore causal relationships cannot be inferred from the resulting analyses. Participants were not stratified by sex, age, body weight or other baseline characteristics and were analysed descriptively as a total population. For summary statistics, continuous variables were presented as either mean (standard deviation [SD]) or median (interquartile range [IQR]). Categorical variables were described by number and proportion of patients in each category. Missing data were not imputed in either study.

For the cross-sectional study, the proportions of patients with 3P-MACE and with composite CVD outcome were measured in the two time periods and reported as n (%). Descriptive statistics for patient demographics, clinical characteristics, HCRU, and medication prescriptions were provided for each 1-year time period. Additionally, patient characteristics and prevalence of 3P-MACE and the composite CVD outcome were stratified by prescription of antidiabetes medication. In the retrospective cohort study, incidence of CVD, including both 3P-MACE and the composite CVD outcome, was reported during the follow-up period and incidence rate estimates were calculated by dividing the number of new CVD events by the person-years (PY) at risk and presented per 1000 PY. Time at risk was calculated from the day after the index date until end of study period, loss to follow-up, death, or time of event. Incidence of CVD was also stratified by patients with and without a history of CVD, with history of CVD defined as the history of the composite CVD outcome (excluding cardiovascular death).

Survival curves were derived for time from the day after the index date to first CVD diagnosis using the Kaplan–Meier method. Participant characteristics and HCRU were reported and stratified by baseline history of CVD and antidiabetes medication prescription. Antidiabetes medication treatment patterns were examined in patients with ≥ 2 years of antidiabetes medication history. Treatment patterns were examined in subgroups defined by history of CVD at baseline. The most recent antidiabetes medications ± 60 days of index date, and 180, 360, 540, and 720 days after index date were reported. Antidiabetes medications were categorised by drug class.

## Results

### Cross-sectional cohort study

#### Demographics and clinical characteristics

In 2014, the study population comprised 292,383 individuals; in 2021 there were 622,531. The mean (SD) age of the study population was 67.8 (12.4) years in 2014 and 69.6 (13.0) years in 2021. In 2014, 61.2% were male and in 2021, 62.5% were male. In 2014, 8444 (2.9%) were newly diagnosed with T2D, with 9300 (1.5%) newly diagnosed in 2021 (Table S1).

In 2014, 2.1% of patients received GLP-1RAs and 0.8% received SGLT-2is; in 2021 these values were 9.2% and 32.3%, respectively (Table S1). Between 2014 and 2021, prescription of biguanide (32.6% vs. 41.6%), dipeptidyl-peptidase 4 inhibitors (DPP-4is; 62.1% vs. 67.6%), and glinides (7.9% vs. 10.1%) increased, whereas the prescription of insulin remained consistent (36.4% vs. 36.7%), and there was decreased prescription of sulfonylureas (33.2% vs. 16.7%), thiazolidinediones (11.9% vs. 6.2%), and alpha-glucosidase inhibitors (24.5% vs. 14.9%). Cardiovascular-related medication prescription patterns also changed from 2014 to 2021 (Table S1). Prescription of lipid-modifying agents increased from 49.1% to 54.7% and prescription of antihypertensives from 67.6% to 70.2%. Antithrombotic medication prescription was 45.2% in 2014 and 46.6% in 2021. Mean HbA_1c_ was 7.2% in both 2014 and 2021 (Table S1). Total cholesterol, triglycerides, high-density lipoproteins (HDLs), low-density lipoproteins (LDLs), and eGFR were similar in 2014 and 2021 (Table S1).

#### CVD outcomes

**Fig. 1:**
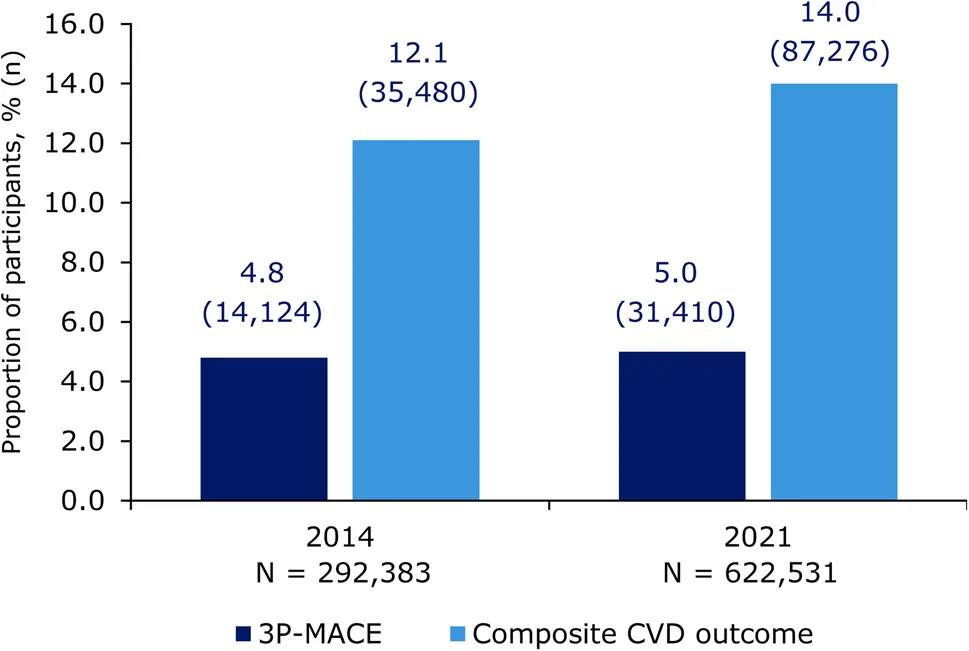
Prevalence of 3P-MACE and composite CVD outcome, over each time period. 3P-MACE was defined as a composite of cardiovascular death, non-fatal MI, and non-fatal stroke. Composite CVD outcome was defined as the outcome of cardiovascular death, fatal/non-fatal MI, fatal/non-fatal stroke, coronary artery disease, heart failure, peripheral artery disease, and atrial fibrillation. 3P-MACE, three-point major adverse cardiovascular event; CVD, cardiovascular disease; MI, myocardial infarction

Prevalence of 3P-MACE was 4.8% (*n* = 14,124) in 2014 and 5.0% (*n* = 31,410) in 2021 (Fig. 1). Prevalence of the composite CVD outcome was 12.1% (*n* = 35,480) in 2014 and 14.0% (*n* = 87,276) in 2021 (Fig. 1). The prevalence of 3P-MACE and the composite CVD outcome stratified by antidiabetes medication is shown in Table 1. In 2014, the lowest prevalence of 3P-MACE and the composite CVD outcome was observed among patients receiving SGLT-2i (1.7% and 5.5%, respectively), whereas the highest values were in those receiving insulin (8.9% and 21.0%). In 2021, the highest prevalence of 3P-MACE and the composite CVD outcome was again observed among those prescribed insulin (8.7% and 22.0%) (Table 1). Prevalence of 3P-MACE in patients taking GLP-1RAs was 2.7% in 2014 and 3.3% in 2021; prevalence of the composite CVD outcome for these patients was 8.3% in 2014 and 11.1% in 2021 (Table 1). HCRU was similar in 2014 and 2021 (Table S2). The total number of outpatient and T2D-related outpatient visits was higher in 2014 than 2021: median (IQR) monthly number of all outpatient visits was 0.56 (0.37–0.79) and outpatient visits related to T2D 0.52 (0.34–0.75) in 2014, versus 0.48 (0.28–0.71) and 0.44 (0.26–0.66), respectively, in 2021. There were no other occurrences of HCRU recorded in this time period.

**Table 1:** Prevalence of 3P-MACE and composite CVD outcome, stratified by type of antidiabetes medication over each time period

Antidiabetes medication	3P-MACE, *n*/*N* (%)		Composite CVD outcome, *n*/*N* (%)	
	2014	2021	2014	2021
Biguanide	2736/95,220 (2.9)	9509/259,192 (3.7)	6337/95,220 (6.7)	21,710/259,192 (8.4)
GLP-1RA	165/6026 (2.7)	1888/57,411 (3.3)	503/6026 (8.3)	6357/57,411 (11.1)
DPP-4i	8778/181,609 (4.8)	21,344/420,731 (5.1)	22,505/181,609 (12.4)	57,763/420,731 (13.7)
SGLT-2i	41/2385 (1.7)	8774/200,932 (4.4)	131/2385 (5.5)	29,740/200,932 (14.8)
Sulfonylureas	3808/97,124 (3.9)	4567/103,985 (4.4)	9468/97,124 (9.7)	11,813/103,985 (11.4)
Glinide	1026/23,201 (4.4)	2934/62,709 (4.7)	2829/23,201 (12.2)	8905/62,709 (14.2)
Thiazolidinedione	1028/34,862 (2.9)	1242/38,287 (3.2)	2480/34,862 (7.1)	3033/38,287 (7.9)
Alpha-GI	2807/71,709 (3.9)	3975/92,536 (4.3)	7936/71,709 (11.1)	11,494/92,536 (12.4)
Imeglimin	NA	3/205 (1.5)	NA	13/205 (6.3)
Insulin	9520/106,501 (8.9)	19,879/228,746 (8.7)	22,376/106,501 (21.0)	50,238/228,746 (22.0)

3P-MACE, three-point major adverse cardiovascular event; alpha-GI, alpha-glucosidase inhibitor; CVD, cardiovascular disease; DPP-4i, dipeptidyl-peptidase 4 inhibitor; GLP-1RA, glucagon-like peptide-1 receptor agonist; NA, not available; SGLT-2i, sodium-glucose cotransporter-2 inhibitor

### Retrospective cohort study

#### Demographics and clinical characteristics

The study population comprised 314,206 individuals: 62,030 had a history of CVD and 252,176 no history of CVD. The overall mean (SD) age of the study population was 70.4 (13.3) years. Mean (SD) age was higher in those with a history of CVD (75.9 [11.1]) than in those with no history of CVD (69.0 [13.4]). Overall, 59.5% were male, with similar proportions in those with and without a history of CVD. In total, 13,163 (4.2%) were newly diagnosed with T2D on or after the index date, with 7.4% of those with a history of CVD and 3.4% with no history of CVD being newly diagnosed (Table S3).

**Fig. 2:**
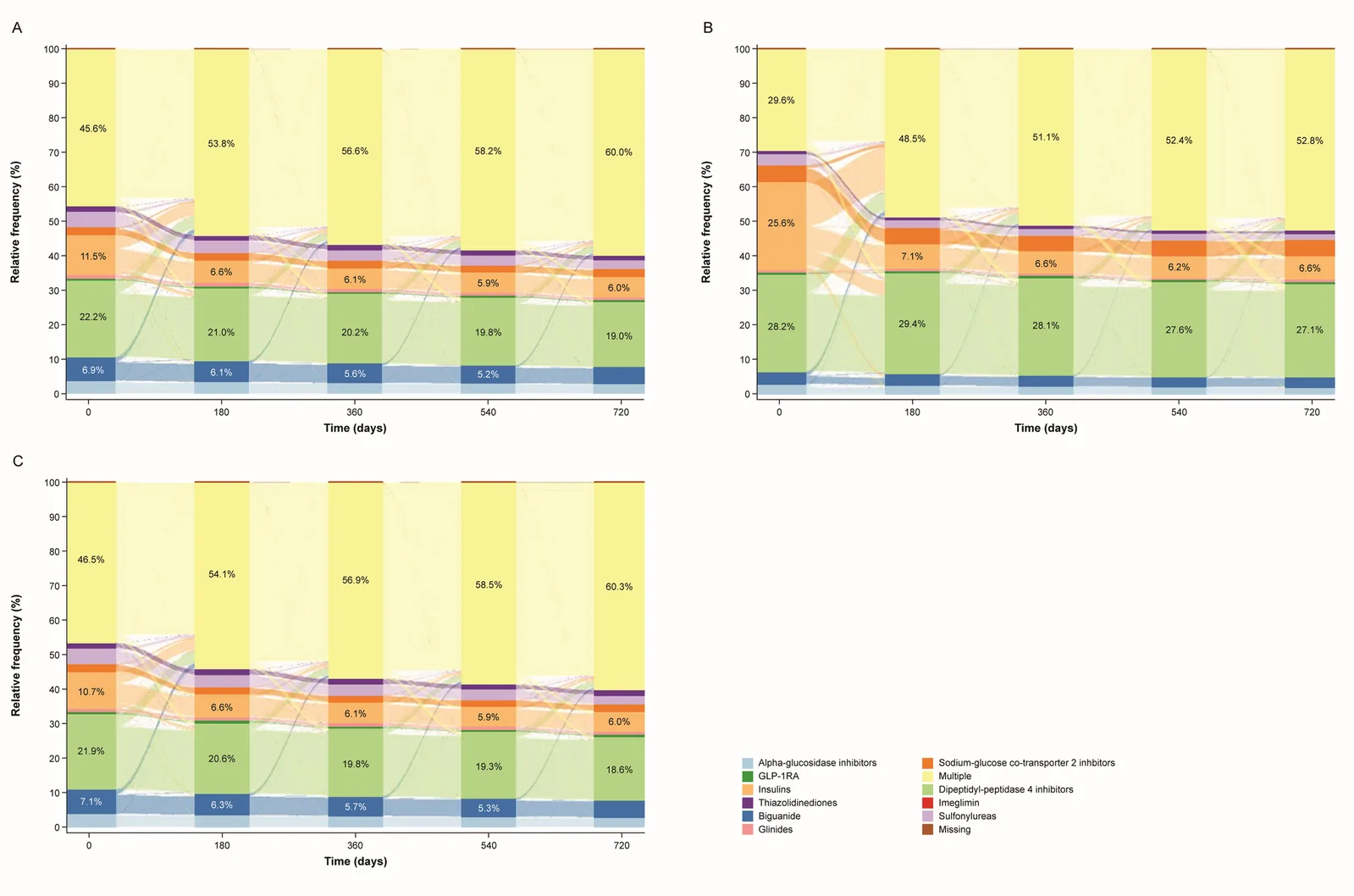
Treatment patterns of antidiabetes medication in the retrospective cohort study. Data are relative proportion of participants (%) taking individual or multiple antidiabetes medications from index date to 720 days post index date. Data are shown for the overall study population (**a**), those with no history of CVD (**b**), and those with history of CVD (**c**). CVD, cardiovascular disease; GLP-1RA, glucagon-like peptide-1 receptor agonist

At baseline, SGLT-2is were prescribed (as monotherapy or part of multiple medication therapy) in 15.9% of patients with T2D with a history of CVD and 10.2% in those with no history of CVD. GLP-1RAs were prescribed to 2.2% of the overall study population, at similar levels for patients with and without a history of CVD. DPP-4is were the most-prescribed antidiabetes medication (55.7% of patients) with similar prescription levels in those with and without a history of CVD. At the index date, 45.6% of patients were prescribed multiple antidiabetes medications. Overall, 22.2% of patients were prescribed a DPP-4i, 11.5% insulin, and 6.9% biguanide as antidiabetes monotherapy (Fig. 2a). The greatest shift in prescribing patterns occurred after the first 180 days, which was the first observational date following the index date. After 180 days, the percentage of participants in the overall study population using multiple antidiabetes medications increased from 45.6% to 53.8%, whereas the percentage of those prescribed insulin decreased from 11.5% to 6.6%. In those with no history of CVD both multiple medication and insulin prescriptions were similar to those in the overall study population (Figs. 2a and S2c). However, in those with a history of CVD, multiple medication prescription was 29.6% at index and 48.5% at 180 days, and insulin prescription was 25.6% at index and 7.1% at 180 days (Fig. 2b). Meanwhile, prescription of DPP-4is or biguanide remained relatively stable between index date and day 180, although DPP-4i prescription was numerically higher in those with a history of CVD. These trends persisted even after 720 days, with treatment patterns showing little variation (Fig. 2).

Cardiovascular-related medication prescription differed for patients with and without a history of CVD (Table S3). In patients with a history of CVD, lipid-modifying agent prescription was 40.5%, antihypertensive prescription 76.6%, and antithrombotic prescription 78.9%. In patients with no history of CVD, lipid-modifying agent prescription was 35.4%, antihypertensive prescription 53.9%, and antithrombotic prescription 33.7%. Hypertension was the most prevalent clinical characteristic other than CVD, followed by hyperlipidaemia, microvascular nephropathy, microvascular retinopathy, dementia, and microvascular neuropathy (Table S3).

Hypertension was more prevalent in patients with a history of CVD than in those without (66.3% vs. 52.9%) (Table S3). Hyperlipidaemia was similar in both groups, present in 41.0% of patients with CVD and 39.6% of patients without CVD. The prevalence of microvascular nephropathy was 6.0% in patients with CVD and 7.0% in those without. Microvascular retinopathy and microvascular neuropathy were slightly more prevalent in the cohort of patients without CVD (7.2% and 4.9%, respectively) than in the cohort with CVD (3.4% and 2.5%, respectively). Lab test results varied between the groups (Table S3); mean HbA_1c_ was lower in patients with a history of CVD than those with no history of CVD (7.08 vs. 7.54). Total cholesterol, triglycerides, HDLs, LDLs, and eGFR were lower in patients with a history of CVD than those without.

#### CVD outcomes

**Fig. 3:**
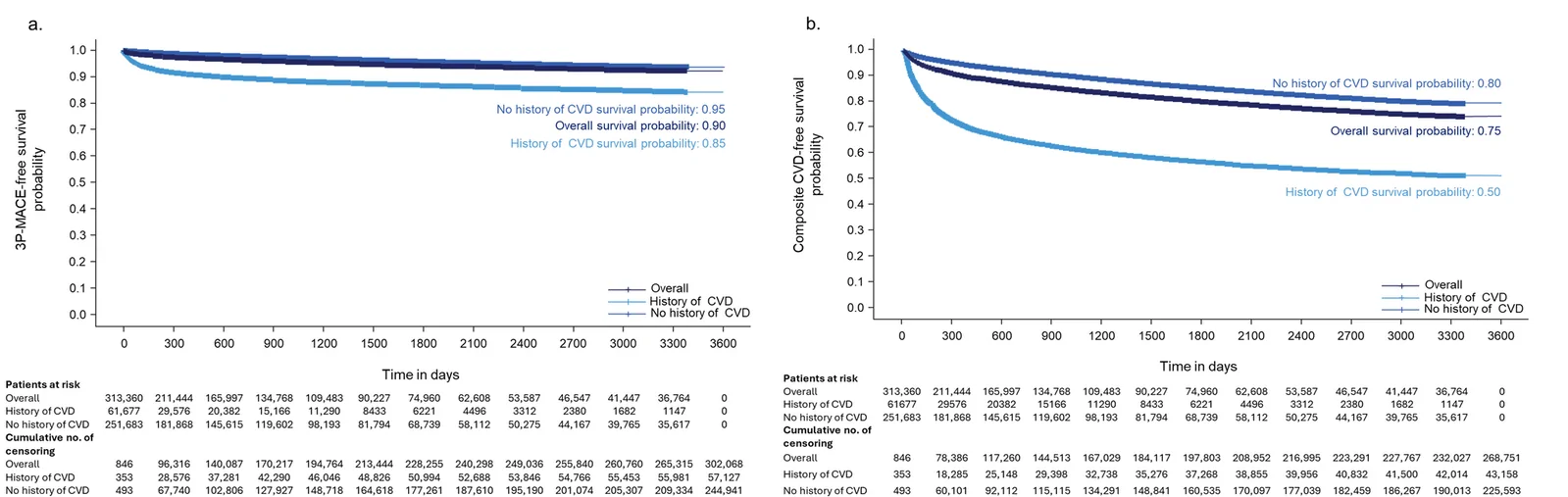
Time to 3P-MACE-free survival (**a**) and composite CVD-free survival (**b**), by history of CVD at baseline. After a maximum of 3600 days of observation, the time to first incidence of 3P-MACE (**a**) dropped to approximately 0.90 for the overall study population, 0.85 for patients with history of CVD at baseline, and 0.95 for patients without history of CVD at baseline. For composite CVD-free survival (**b**), probability dropped to 0.75, 0.50, and 0.80, respectively. Patients who died, or who did not experience the study event prior to the study end date, were censored at study end. Patients who were lost to follow-up were censored at the time of their last event data. 3P-MACE, three-point major adverse cardiovascular event; CVD, cardiovascular disease

In total, 12,138 3P-MACE events were recorded during the study. For the total study population, the incidence rate of 3P-MACE per 1000 PY was 12.8 (95% CI 12.6–13.0) (Table 2). For patients with a history of CVD the incidence rate per 1000 PY was 46.6 (95% CI 45.3–48.0); for patients without a history of CVD it was 8.6 (8.4–8.8). In total, 45,455 composite CVD outcome events were recorded during the study. For the total study population, the incidence rate of the composite CVD outcome per 1000 PY was 47.9 (95% CI 47.4–48.3) (Table 2). For patients with history of CVD it was 179.5 (177.0–182.1); for patients with no history of CVD it was 31.5 (31.1–31.8). Time-to-event analyses showed that overall probability of 3P-MACE-free survival was 0.10 over 3600 days; 0.15 in patients with a history of CVD and 0.05 in patients with no history of CVD (Fig. 3a). The overall probability of the composite CVD-free survival was 0.25; 0.50 in patients with a history of CVD and 0.20 for patients without (Fig. 3b). Patients with a history of CVD had more frequent hospitalisations than those without a history of CVD (median 0.14 vs. 0.03), greater median length of stay (1.53 vs. 0.23 days), and more CVD-related outpatient visits per month (median 0.35 vs. 0.00) (Table S4).

**Table 2:** Incidence of 3P-MACE and composite CVD outcome in adults with T2D in Japan overall and stratified by history of CVD at baseline

	Study population *N* = 314,206	T2D cohort with CVD *N* = 62,030	T2D cohort without CVD *N* = 252,176
*3P-MACE*			
Total follow-up period (person-years)	949,875	105,116	844,759
Number of events	12,138	4903	7235
Incidence rate, per 1000 person-years (95% CI)	12.8 (12.6–13.0)	46.6 (45.4–48.0)	8.6 (8.4–8.8)
*Composite CVD outcome*			
Total follow-up period (person-years)	949,875	105,116	844,759
Number of events	45,455	18,872	26,583
Incidence rate, per 1000 person-years (95% CI)	47.9 (47.4–48.3)	179.5 (177.0–182.1)	31.5 (31.1–31.9)

3P-MACE, three-point major adverse cardiovascular event; CI, confidence interval; CVD, cardiovascular disease; T2D, type 2 diabetes

## Discussion

In this study, which comprised a cross-sectional study and a retrospective cohort study of adults in Japan with T2D, the prevalence of both 3P-MACE and composite CVD outcome was almost identical in 2014 and 2021; the incidence rates of 3P-MACE and composite CVD outcome were 5.4 and 5.7 times higher, respectively, in patients with versus without a history of CVD. These data indicate that Japanese individuals with T2D and a history of CVD represent an at-risk population; therefore, treatment focusing on reducing CVD risk could be beneficial. Accordingly, prescription of glucose-lowering medications with known cardiovascular benefits, such as GLP-1RAs and SGLT-2is, was observed in this study to increase from 2014 to 2021.

Previous studies have assessed the incidence rates of cardiovascular events in people with T2D. A study of patients with T2D in Japan receiving DPP-4is found the incidence rate of MI requiring hospitalisation to be 2.35 per 1000 PY (95% CI 2.18–2.53) and that of stroke requiring hospitalisation to be 11.84 per 1000 PY (95% CI 11.46–12.23) [24]. These incidence rates, when taken together (approximately 14 per 1000 PY), were similar to that observed for 3P-MACE in this study (approximately 13 per 1000 PY), suggesting that our findings are reflective of the overall Japanese T2D population. In a UK-based study of people with T2D, the incidence rates of 3P-MACE were similar, compared with the present study, at 14.0 per 1000 PY for individuals without established CVD and 49.6 per 1000 PY for those with CVD [25], highlighting the global nature of CVD in T2D. A study that utilised the MDV database to assess risk of CVD development found that patients also had an increased risk of all-cause mortality and other cardiovascular events, when patients had prior history of cardiorenal disease [26]. In a further study of CVD risk in Japanese adults, 8-year cumulative incidence rates of CVD in patients with T2D with and without a history of CVD were 21.5% and 7.2%, respectively [27]; these slightly higher rates could be explained by differences in study populations and definitions of CVD. CVD in this study was defined as a composite of CAD, hospital admission for HF, cerebrovascular disease, and PAD, whereas it is defined in the present study as a composite of MI, stroke, CAD, HF, PAD, and AF. However, when the present study is taken together with existing evidence, it indicates that a prior history of CVD increases risk of future CVD events in Japanese adults with T2D; as such, medications for treatment of T2D should also aim to reduce cardiovascular risk in this population. Atrial fibrillation was included in the composite CVD outcome of this study. Atrial fibrillation is highly prevalent in older Japanese adults [28]. Therefore, the present analysis may inflate the prevalence of composite CVD compared with other similar analyses which have not included atrial fibrillation in their composite CVD endpoint. However, the inclusion of atrial fibrillation within the composite CVD outcome is justified as the data presented compare between two different timepoints (2014 and 2021) and therefore meaningful within trial comparisons can be made. Additionally, we evaluated 3P-MACE, a standardised outcome, alongside the composite CVD outcome.

In both component studies, demographics and characteristics such as age and sex were similar to those in other studies investigating T2D in Japanese populations [24, 26, 27]. In the retrospective cohort study, people with a history of CVD were older than those with no history of CVD, in line with evidence indicating that age is a risk factor for CVD [29]. In a previous cross-sectional study, CVD prevalence was 37.3% in patients aged ≥ 20 years with T2D in 2018–2019 [3]. However, owing to differences in definition of composite CVD (the previous study used cerebrovascular disease, coronary heart disease, HF, cardiac arrhythmia or conduction abnormalities, aortic disease, PAD, or CAD), direct comparison between studies is difficult. Additionally, the present study included data from inpatient facilities only whereas the previous study took data from both inpatient and outpatient facilities, which could alter the composition of the study population.

DPP-4is were the most prescribed antidiabetes medication in both component studies, consistent with other studies of populations with T2D in Japan [23, 30]. In both component studies, insulin prescription was associated with greater CVD prevalence and incidence, potentially as people with T2D receiving insulin are likely to have more advanced disease, with increased risk of CVD [31]. In a retrospective database study of Japanese adults with T2D between 2017 and 2019, SGLT-2is and GLP-1RAs were prescribed to 12.6% and 7.0% of the study population [32], respectively, in line with the increased prescriptions of these medications from 2014 to 2021 observed in the present cross-sectional study. Although the cardiovascular health effects of these drugs would be expected to lower CVD risk, our results did not indicate this despite a greater number of patients being prescribed GLP-1RAs and SGLT‑2is in 2021 than 2014. However, it should be noted that the lack of increase in CVD may indicate a stable prevalence in an aging population, where a rise in CVD would typically be expected [33]. Additionally, a longer time period may be needed to observe a reduction in 3P-MACE and composite CVD outcome prevalence arising from increased prescription of these medications. Furthermore, since no adjustment for confounding factors, such as multivariate analysis or propensity score matching, was performed, the findings should be interpreted with caution. These findings therefore highlight the need for additional analysis particularly addressing the prevalence of 3P-MACE and CVD across age groups.

The MDV database has previously provided insight into HCRU and costs for the Japanese T2D population. SGLT-2i prescription has previously been shown to increase medication costs for Japanese individuals with T2D but also to result in less frequent hospitalisation and a shorter stay [34]. However, in this study, HCRU remained consistent between 2014 and 2021, although there was a decrease in both outpatient and T2D-related outpatient visits. It is possible that the COVID-19 pandemic contributed to the reduced number of outpatient visits during this time [35]. Those with a history of CVD utilised health care resources more frequently than those without a history of CVD. Previously published data have also shown that SGLT-2i versus DPP-4i prescriptions were associated with a significant reduction in HF and all-cause mortality in Japanese adults with T2D and without a history of CVD [24]. Additionally, as part of a global real-world retrospective study, SGLT-2i use was associated with a reduced risk of cardiovascular events across a broad range of patient characteristics [36]. Recent cardiovasvular outcome trials have shown that GLP-1RAs and SGLT-2is may provide cardiovascular health benefits in individuals with T2D with established CVD [1, 37, 38]; as such, it is to be expected that prescriptions of GLP-1RAs and SGLT-2is would increase from 2014 to 2021. However, despite the increased prescription of these medications, the prevalence of CVD in these populations also numerically increased. It may be the case that additional follow-up is needed to determine the long-term impact of increased GLP-1RA and SGLT-2i prescriptions. Further cardiovascular outcome trials and real-world evidence studies are therefore likely needed to determine the effect these medications have in Japanese adults with T2D and CVD.

This study has some limitations. No conclusions can be drawn on associations between any of the characteristics explored, as this was a descriptive, non-interventional study, and neither comparative or sensitivity analyses were performed. Therefore causal relationships cannot be inferred from the resulting analyses. In the cross-sectional study, medication changes over time were not considered. Therefore, a relationship between CVD events and medication changes over time cannot be inferred from these data. Additionally, disease severity was not assessed as part of the data collected by the JMDC database, therefore changes in treatment over time were unable to be categorised by disease severity. Only 10% of medical institutions provided lab test data; therefore, data may not accurately represent the total study population, however, due to the size of the trial population the sample size is adequate to support meaningful descriptive analysis. The MDV database provides data from approximately 450 large or medium acute-care hospitals only, and not from other healthcare settings; this is potentially reflected in the lower frequency of hospital visits by patients after the index date in the retrospective cohort analysis. As such the incidence of newly diagnosed T2D in this acute care setting may not be reflective of the general population despite the current study population not being limited by age, disease, or socio-economic status per the inclusion criteria. Furthermore, the fact that routine clinical management in smaller or rural health care facilities is unrepresented introduces selection bias towards those with more moderate or urgent health care needs or those with advanced stage T2D. Taken together, these data could be considered generalisable to a large portion of individuals in Japan living with diabetes but may be biased towards populations centralised around larger population areas.

In conclusion, this study provides insights into the prevalence and incidence of both 3P-MACE and composite CVD outcome in individuals with T2D in Japan. These data indicate that whereas prescription patterns for diabetes medications with cardiovascular benefit are increasing, CVD risk remained largely unchanged in the overall Japanese population with T2D, although there was a slight decrease in outpatient visits and T2D-related outpatient visits from 2014 to 2021. These data may provide novel insights into the treatment patterns of T2D in Japan and help to inform real-world decision making in the management of T2D with concomitant CVD in the Japanese population.

## Supplementary Information

Below is the link to the electronic supplementary material.

Supplementary Material 1

## Data Availability

The claims database that supports the findings of this study is available from Medical Data Vision Co., Ltd. but was used under licence for the current study; therefore, restrictions apply, and the data are not publicly available. For inquiries about access to the database used in this study, please contact MDV (https://www.mdv.co.jp/; email address, ebm_sales@mdv.co.jp).
