# Correction: Retrospective real-world database study to examine the prevalence and incidence of cardiovascular diseases and medication prescription in Japanese patients with type 2 diabetes

**DOI:** 10.1007/s13340-026-00919-0

**Published:** 2026-08-21

**Authors:** Mitsuhisa Komatsu, Hiroshi Kobayashi, Satoshi Tsuboi, Hirotaka Watada

**Affiliations:** 1 Division of Diabetes, Endocrinology and Metabolism, Department of Internal Medicine, Shinshu University School of Medicine, Asahi 3–1-1, Matsumoto, Nagano 390–8621 Japan; 2 Cardiovascular and Emerging Therapy Areas, Medical Affairs Department, CMRQ Development Division, Novo Nordisk Pharma Ltd, Tokyo, Japan; 3 RWE Group, SPVD Department, CMRQ Development Division, Novo Nordisk Pharma Ltd, Tokyo, Japan; 4 Department of Metabolism and Endocrinology, Juntendo University Graduate School of Medicine, Tokyo, Japan

**Correction to: Diabetology International (2026) 17:31** **https://doi.org/10.1007/s13340-026-00877-7**

In this article, Fig(s) 2 and 3 appeared incorrectly and have now been corrected in the original publication. For completeness and transparency, the old incorrect and corrected versions are displayed below.

**Incorrect Version**

**Fig. 2:**
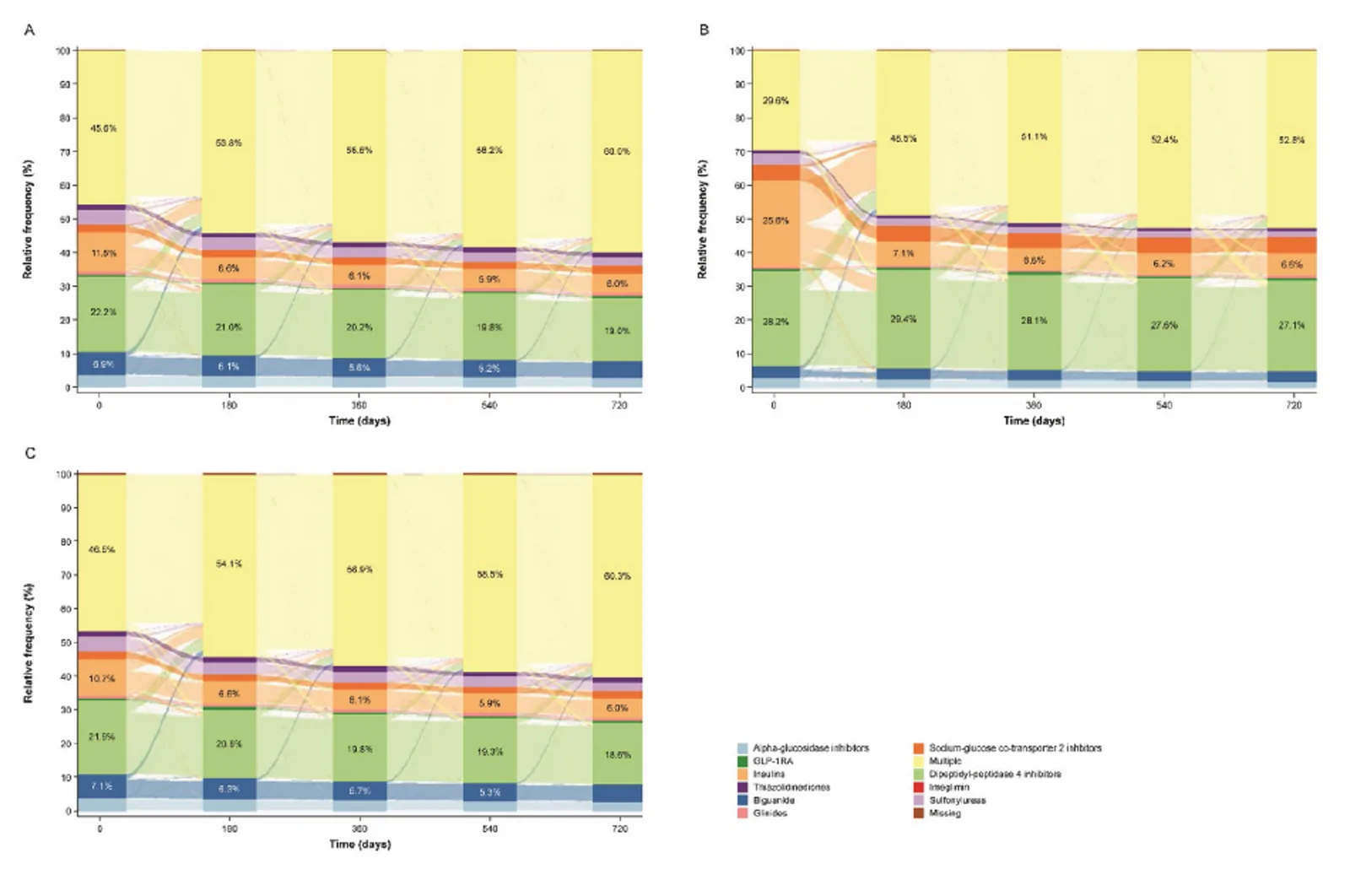
Treatment patterns of antidiabetes medication in the retrospective cohort study. Data are relative proportion of participants (%) taking individual or multiple antidiabetes medications from index date to 720 days post index date. Data are shown for the overall study population (**a**), those with no history of CVD (**b**), and those with history of CVD

**Fig. 3:**
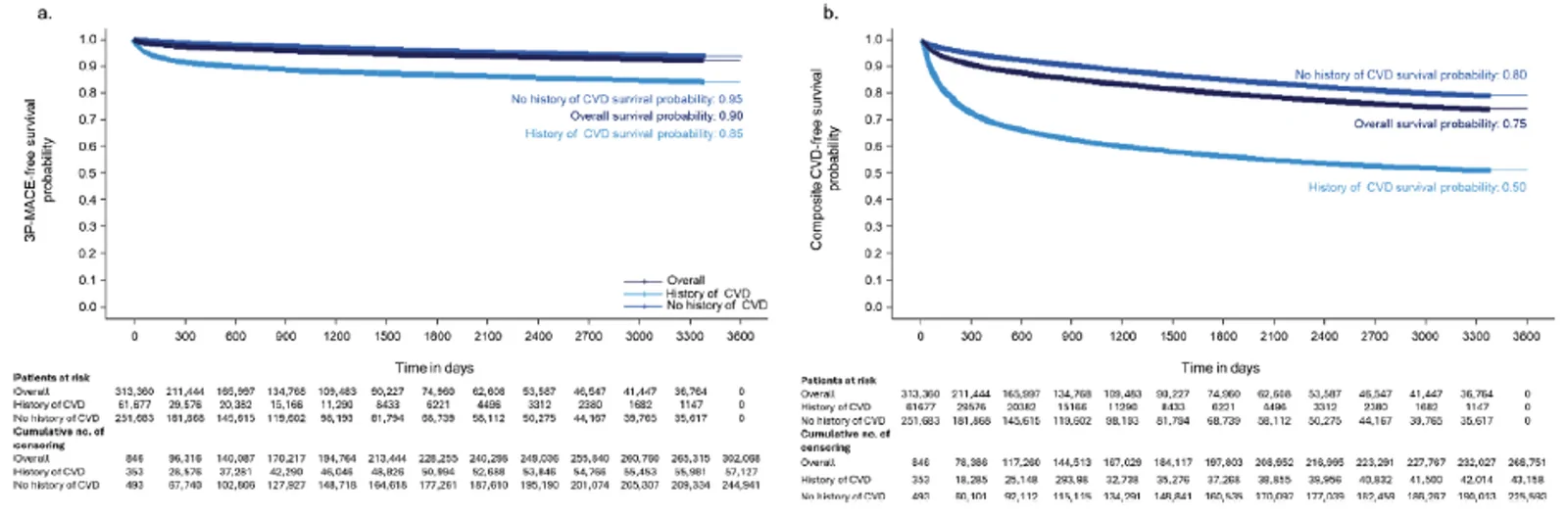
Time to 3P-MACE-free survival (**a**) and composite CVD-free survival (**b**), by history of CVD at baseline. After a maximum of 3600 days of observation, the time to first incidence of 3P-MACE (**a**) dropped to approximately 0.9 for the overall study population, 0.85 for patients with history of CVD at baseline, and 0.95 for patients without history of CVD at baseline. For composite CVD-free survival (**b**), probability dropped to 0.75, 0.50, and 0.80, respectively. Patients who died, or who did not experience the study event prior to the study end date, were censored at study end. Patients who were lost to follow-up were censored at the time of their last event data. 3P-MACE, three-point major adverse cardiovascular event; CVD, cardiovascular disease; GLP-1RA, glucagon-like peptide-1 receptor agonist

**Correct Version**.

The original article has been corrected.

**Fig. 2:**
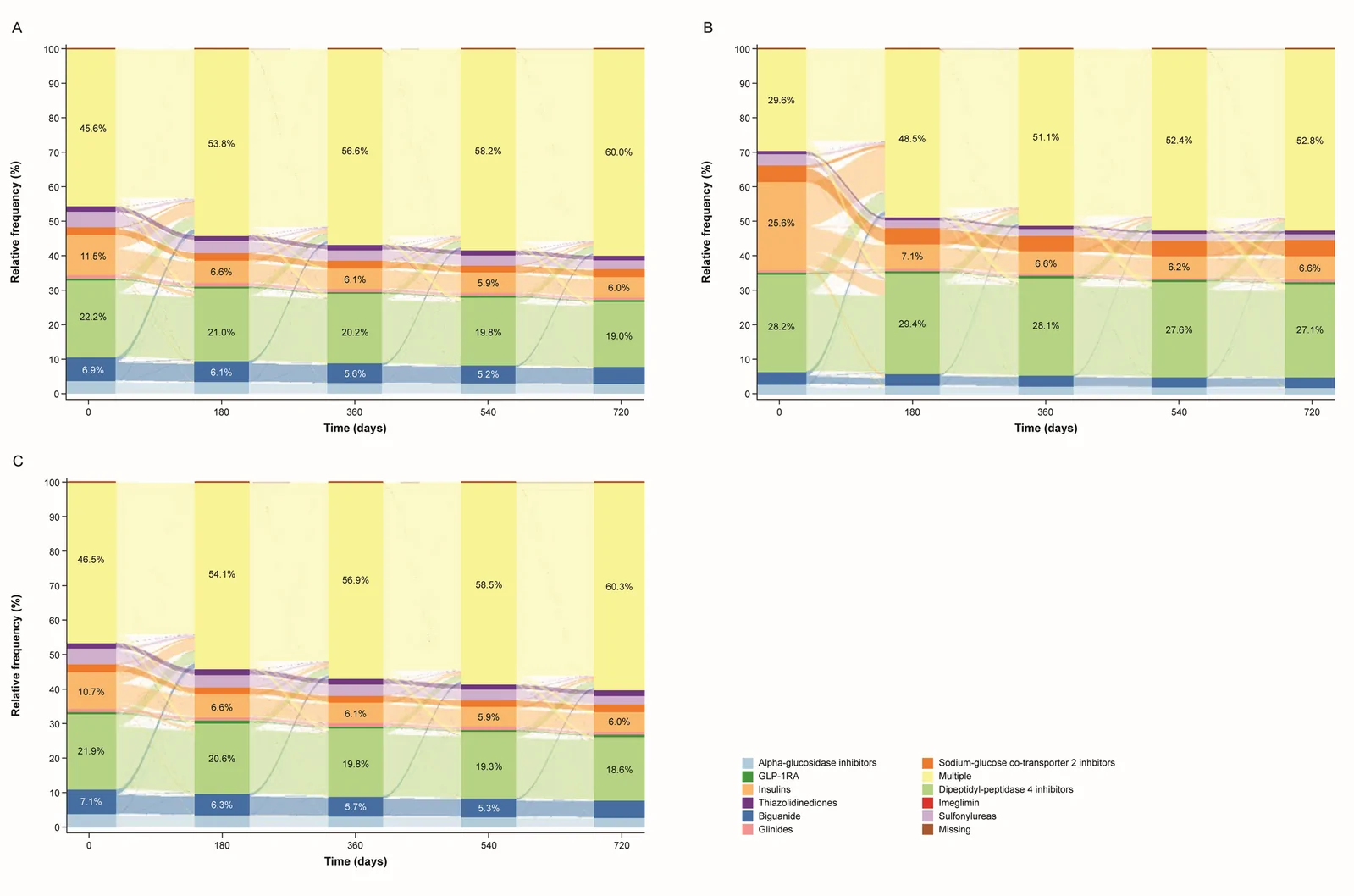
Treatment patterns of antidiabetes medication in the retrospective cohort study. Data are relative proportion of participants (%) taking individual or multiple antidiabetes medications from index date to 720 days post index date. Data are shown for the overall study population (**a**), those with no history of CVD (**b**), and those with history of CVD

**Fig. 3:**
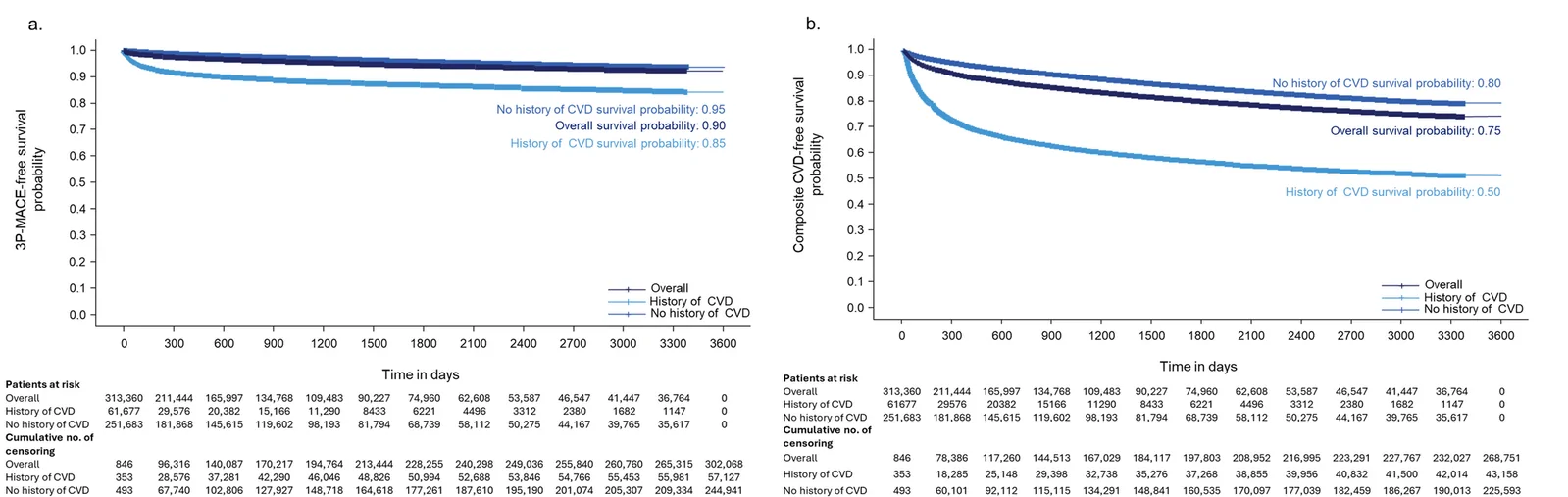
Time to 3P-MACE-free survival (**a**) and composite CVD-free survival (**b**), by history of CVD at baseline. After a maximum of 3600 days of observation, the time to first incidence of 3P-MACE (**a**) dropped to approximately 0.9 for the overall study population, 0.85 for patients with history of CVD at baseline, and 0.95 for patients without history of CVD at baseline. For composite CVD-free survival (**b**), probability dropped to 0.75, 0.50, and 0.80, respectively. Patients who died, or who did not experience the study event prior to the study end date, were censored at study end. Patients who were lost to follow-up were censored at the time of their last event data. 3P-MACE, three-point major adverse cardiovascular event; CVD, cardiovascular disease; GLP-1RA, glucagon-like peptide-1 receptor agonist

