# Supplementary material for: Retrospective real-world database study to examine the prevalence and incidence of cardiovascular diseases and medication prescription in Japanese patients with type 2 diabetes
Source: Diabetol Int. 2026 Mar 17;17(2):31. doi: 10.1007/s13340-026-00877-7 (PMC12992755; doi:10.1007/s13340-026-00877-7)
Supplement: Supplementary file 1 — Supplementary Material 1 [file 13340_2026_877_MOESM1_ESM.docx]

# Retrospective real-world database study to examine the prevalence and incidence of cardiovascular diseases and medication prescription in Japanese patients with type 2 diabetes

# Supplementary materials

Mitsuhisa Komatsu^1^, Hiroshi Kobayashi^2^, Satoshi Tsuboi^3^, Hirotaka Watada^4^

^1^Division of Diabetes, Endocrinology and Metabolism, Department of Internal Medicine, Shinshu University School of Medicine, Nagano, Japan; ^2^Cardiovascular and Emerging Therapy Areas, Medical Affairs Department, CMRQ Development Division, Novo Nordisk Pharma Ltd, Tokyo, Japan; ^3^RWE Group, SPVD Department, CMRQ Development Division, Novo Nordisk Pharma Ltd, Tokyo, Japan; ^4^Department of Metabolism and Endocrinology, Juntendo University Graduate School of Medicine, Tokyo, Japan

## Corresponding author:

## Mitsuhisa Komatsu

## Division of Diabetes, Endocrinology and Metabolism, Department of Internal Medicine, Shinshu University School of Medicine, Asahi 3–1-1, Matsumoto, Japan 390–8621

## Email: [mitsuk@shinshu-u.ac.jp](mailto:mitsuk@shinshu-u.ac.jp)

**Contents**

[Table S1 Cross-sectional study baseline characteristics and medication prescription over each time period 3](#_Toc213320194)

[Table S2 Cross-sectional study baseline HCRU over each time period 5](#_Toc213320195)

[Table S3 Demographic characteristics and medication prescription of retrospective cohort study population stratified by history of CVD at baseline 6](#_Toc213320196)

[Table S4 HCRU of study population and cohorts overall and stratified by history of CVD at baseline in the retrospective cohort study 8](#_Toc213320197)

[Fig. S1 Study design for cross-sectional (a) and retrospective cohort study (b) 9](#_Toc213320198)

Table S1 Cross-sectional study baseline characteristics and medication prescription over each time period

|  | **2014  N = 292,383** | **2021  N = 622,531** |
| --- | --- | --- |
| **Demographics** | | |
| Age, mean (SD) | 67.8 (12.4) | 69.6 (13.0) |
| Male, n (%) | 178,930 (61.2) | 389,042 (62.5) |
| Newly diagnosed T2D, n (%) | 8444 (2.9) | 9300 (1.5) |
| **Antidiabetes medication, n (%)** | | |
| DPP-4i | 181,609 (62.1) | 420,731 (67.6) |
| Insulin | 106,501 (36.4) | 228,746 (36.7) |
| Sulfonylureas | 97,124 (33.2) | 103,985 (16.7) |
| Biguanide | 95,220 (32.6) | 259,192 (41.6) |
| alpha-GI | 71,709 (24.5) | 92,536 (14.9) |
| Thiazolidinedione | 34,862 (11.9) | 38,287 (6.2) |
| Glinide | 23,201 (7.9) | 62,709 (10.1) |
| GLP-1RA | 6026 (2.1) | 57,411 (9.2) |
| SGLT-2i | 2385 (0.8) | 200,932 (32.3) |
| Imeglimin | 0 | 205 (0.0) |
| **Other medications, n (%)** | | |
| Lipid-modifying agents | 143,495 (49.1) | 340,749 (54.7) |
| Antihypertensives | 197,783 (67.6) | 436,896 (70.2) |
| Antithrombotic agents | 132,061 (45.2) | 290,222 (46.6) |
| **Clinical characteristics other than CVD, n (%)** | | |
| Hypertension | 202,596 (69.3) | 422,722 (67.9) |
| Hyperlipidaemia | 177,034 (60.5) | 376,339 (60.5) |
| Microvascular complications | 100,025 (34.2) | 189,545 (30.4) |
| Retinopathy | 53,042 (18.1) | 87,160 (14.0) |
| Nephropathy | 43,076 (14.7) | 105,536 (17.0) |
| Neuropathy | 35,103 (12.0) | 54,291 (8.7) |
| Dementia | 11,137 (3.8) | 32,116 (5.2) |
| **Laboratory test results** | | |
| HbA_1c_ | | |
| n (%) | 35,322 (12.1) | 70,877 (11.4) |
| Mean (SD), % | 7.21 (1.253) | 7.24 (1.205) |
| Total cholesterol | | |
| n (%) | 24,826 (8.5) | 47,904 (7.7) |
| Mean (SD), mg/dL | 178.47 (36.381) | 178.02 (38.957) |
| Triglycerides | | |
| n (%) | 30,850 (10.6) | 62,847 (10.1) |
| Mean (SD), mg/dL | 145.30 (96.285) | 150.71 (101.885) |
| LDL-cholesterol | | |
| n (%) | 26,678 (9.1) | 53,992 (8.7) |
| Mean (SD), mg/dL | 104.71 (28.767) | 101.53 (30.315) |
| HDL-cholesterol | | |
| n (%) | 29,434 (10.1) | 58,670 (9.4) |
| Mean (SD), mg/dL | 52.48 (14.749) | 53.94 (15.391) |
| eGFR | | |
| n (%) | 34,031 (11.6) | 73,284 (11.8) |
| Mean (SD), mL/min/1.73 m^2^ | 68.88 (24.757) | 65.01 (23.990) |

alpha-GI, alpha-glucosidase inhibitor; CVD, cardiovascular disease; DPP-4i, dipeptidyl-peptidase 4 inhibitor; eGFR, estimated glomerular filtration rate; GLP-1RA, glucagon-like peptide-1 receptor agonist; HbA_1c_, glycated haemoglobin; HDL, high-density lipoprotein; LDL, low-density lipoprotein; SD, standard deviation; SGLT-2i, sodium-glucose cotransporter-2 inhibitor; T2D, type 2 diabetes.

## Table S2 Cross-sectional study baseline HCRU over each time period

| **HCRU, per patient per month** | **2014**  **N = 292,383** | **2021**  **N = 622,531** |
| --- | --- | --- |
| Outpatient visits | 0.56 (0.37–0.79) | 0.48 (0.28–0.71) |
| Hospitalisations via ER | 0.00 (0.00–0.00) | 0.00 (0.00–0.00) |
| Hospitalisations | 0.00 (0.00–0.10) | 0.00 (0.00–0.12) |
| Length of stay in hospital | 0.00 (0.00–1.15) | 0.00 (0.00–1.42) |
| CVD-related outpatient visits | 0.00 (0.00–0.50) | 0.00 (0.00–0.42) |
| T2D-related outpatient visits | 0.52 (0.34–0.75) | 0.44 (0.26–0.66) |
| CVD-related hospitalisations via ER | 0.00 (0.00–0.00) | 0.00 (0.00–0.00) |
| T2D-related hospitalisations via ER | 0.00 (0.00–0.00) | 0.00 (0.00–0.00) |
| CVD-related hospitalisations | 0.00 (0.00–0.00) | 0.00 (0.00–0.00) |
| T2D-related hospitalisations | 0.00 (0.00–0.00) | 0.00 (0.00–0.00) |
| CVD-related length of stay in hospital | 0.00 (0.00–0.00) | 0.00 (0.00–0.00) |
| T2D-related length of stay in hospital | 0.00 (0.00–0.00) | 0.00 (0.00–0.00) |
| CVD-related lab tests | 0.00 (0.00–0.00) | 0.00 (0.00–0.00) |
| T2D-related lab tests | 0.00 (0.00–0.00) | 0.00 (0.00–0.00) |

Data are median (IQR). Median values were originally calculated to up to four decimal places; no difference was assumed where values are 0.00.

CVD, cardiovascular disease; ER, emergency room; HCRU, healthcare resource utilisation; IQR, interquartile range; T2D, type 2 diabetes.

Table S3 Demographic characteristics and medication prescription of retrospective cohort study population stratified by history of CVD at baseline

|  | **Study population  N = 314,206** | **T2D cohort with CVD  N = 62,030** | **T2D cohort without CVD  N = 252,176** |
| --- | --- | --- | --- |
| **Basic demographics** | | | |
| Age, mean (SD) | 70.4 (13.3) | 75.9 (11.1) | 69.0 (13.4) |
| Male sex, n (%) | 186,805 (59.5) | 38,370 (61.9) | 148,435 (58.9) |
| Newly diagnosed T2D, n (%) | 13,163 (4.2) | 4579 (7.4) | 8584 (3.4) |
| **History of CVD, n (%)** | | | |
| History of CVD | 62,030 (19.7) | 62,030 (100.0) | 0 (0.0) |
| History of 3P-MACE | 24,219 (7.7) | 24,219 (39.0) | 0 (0.0) |
| **Antidiabetes medication, n (%)** | | | |
| DPP-4i | 175,066 (55.7) | 35,154 (56.7) | 139,912 (55.5) |
| Insulin | 134,738 (42.9) | 34,234 (55.2) | 100,504 (39.9) |
| Biguanide | 78,999 (25.1) | 9862 (15.9) | 69,137 (27.4) |
| Sulfonylureas | 57,317 (18.2) | 8469 (13.7) | 48,848 (19.4) |
| Alpha-GI | 43,195 (13.7) | 7231 (11.7) | 35,964 (14.3) |
| SGLT-2i | 35,549 (11.3) | 9887 (15.9) | 25,662 (10.2) |
| Thiazolidinedione | 19,360 (6.2) | 2211 (3.6) | 17,149 (6.8) |
| Glinide | 18,047 (5.7) | 3618 (5.8) | 14,429 (5.7) |
| GLP-1RA | 6852 (2.2) | 1155 (1.9) | 5697 (2.3) |
| Imeglimin | 40 (0.0) | 5 (0.0) | 35 (0.0) |
| **Other medications, n (%)** | | | |
| Lipid-modifying agents | 114,385 (36.4) | 25,134 (40.5) | 89,251 (35.4) |
| Antihypertensives | 183,585 (58.4) | 47,541 (76.6) | 136,044 (53.9) |
| Antithrombotic agents | 134,065 (42.7) | 48,971 (78.9) | 85,094 (33.7) |
| **Clinical characteristics other than CVD, n (%)** | | | |
| Hypertension | 174,533 (55.5) | 41,147 (66.3) | 133,386 (52.9) |
| Hyperlipidaemia | 125,201 (39.8) | 25,423 (41.0) | 99,778 (39.6) |
| Microvascular | 46,370 (14.8) | 6383 (10.3) | 39,987 (15.9) |
| Retinopathy | 20,224 (6.4) | 2094 (3.4) | 18,130 (7.2) |
| Nephropathy | 21,407 (6.8) | 3697 (6.0) | 17,710 (7.0) |
| Neuropathy | 13,778 (4.4) | 1543 (2.5) | 12,235 (4.9) |
| Dementia | 16,823 (5.4) | 5175 (8.3) | 11,648 (4.6) |
| **Laboratory test results** | | | |
| HbA_1c_ |  |  |  |
| n (%) | 36,370 (11.6) | 5606 (9.0) | 30,764 (12.2) |
| Mean (SD), % | 7.47 (1.708) | 7.08 (1.414) | 7.54 (1.747) |
| Total cholesterol |  |  |  |
| n (%) | 26,009 (8.3) | 4497 (7.2) | 21,512 (8.5) |
| Mean (SD), mg/dL | 184.40 (42.7) | 175.34 (43.4) | 186.29 (42.3) |
| Triglycerides |  |  |  |
| n (%) | 30,481 (9.7) | 5060 (8.2) | 25,421 (10.1) |
| Mean (SD), mg/dL | 152.89 (141.4) | 134.02 (92.6) | 156.65 (140.0) |
| LDL-cholesterol |  |  |  |
| n (%) | 25,297 (8.1) | 4302 (6.9) | 20,995 (8.3) |
| Mean (SD), mg/dL | 108.84 (33.5) | 103.90 (35.4) | 109.85 (33.0) |
| HDL-cholesterol |  |  |  |
| n (%) | 27,498 (8.8) | 4694 (7.6) | 22,804 (9.0) |
| Mean (SD), mg/dL | 53.06 (15.6) | 50.20 (15.8) | 53.64 (15.5) |
| eGFR |  |  |  |
| n (%) | 40,273 (12.8) | 6447 (10.4) | 33,826 (13.4) |
| Mean (SD), mL/min/1.73 m^2^ | 67.82 (25.4) | 58.96 (25.4) | 69.51 (25.0) |

3P-MACE, three-point major adverse cardiovascular event; alpha-GI, alpha-glucosidase inhibitor; CVD, cardiovascular disease; DPP-4i, dipeptidyl-peptidase 4 inhibitor; eGFR, estimated glomerular filtration rate; GLP-1RA, glucagon-like peptide-1 receptor agonist; HbA_1c_, glycated haemoglobin; HDL, high-density lipoprotein; LDL, low-density lipoprotein; SD, standard deviation; SGLT-2i, sodium-glucose cotransporter-2 inhibitor; T2D, type 2 diabetes.

### Table S4 HCRU of study population and cohorts overall and stratified by history of CVD at baseline in the retrospective cohort study

| **HCRU, per patient per month** | **Study population**  **N = 314,206** | **T2D cohort with CVD**  **N = 62,030** | **T2D cohort without CVD**  **N = 252,176** |
| --- | --- | --- | --- |
| Outpatient visits | 0.49 (0.16–0.76) | 0.48 (0.06–0.83) | 0.49 (0.19–0.74) |
| Hospitalisations via ER | 0.00 (0.00–0.03) | 0.02 (0.00–0.18) | 0.00 (0.00–0.01) |
| Hospitalisations | 0.04 (0.00–0.17) | 0.14 (0.03–0.63) | 0.03 (0.00–0.12) |
| Length of stay in hospital | 0.37 (0.00–2.06) | 1.53 (0.36–7.78) | 0.23 (0.00–1.39) |
| CVD-related outpatient visits | 0.00 (0.00–0.37) | 0.35 (0.03–0.77) | 0.00 (0.00–0.18) |
| T2D-related outpatient visits | 0.39 (0.06–0.68) | 0.32 (0.02–0.75) | 0.40 (0.08–0.66) |
| CVD-related hospitalisations via ER | 0.00 (0.00–0.00) | 0.00 (0.00–0.05) | 0.00 (0.00–0.00) |
| T2D-related hospitalisations via ER | 0.00 (0.00–0.00) | 0.00 (0.00–0.00) | 0.00 (0.00–0.00) |
| CVD-related hospitalisations | 0.00 (0.00–0.00) | 0.02 (0.00–0.17) | 0.00 (0.00–0.00) |
| T2D-related hospitalisations | 0.00 (0.00–0.00) | 0.00 (0.00–0.00) | 0.00 (0.00–0.00) |
| CVD-related length of stay in hospital | 0.00 (0.00–0.00) | 0.09 (0.00–1.68) | 0.00 (0.00–0.00) |
| T2D-related length of stay in hospital | 0.00 (0.00–0.00) | 0.00 (0.00–0.00) | 0.00 (0.00–0.00) |
| CVD-related lab tests | 0.00 (0.00–0.00) | 0.00 (0.00–0.00) | 0.00 (0.00–0.00) |
| T2D-related lab tests | 0.00 (0.00–0.00) | 0.00 (0.00–0.00) | 0.00 (0.00–0.00) |

Data are median (IQR). Median values were originally calculated to up to four decimal places; no difference was assumed where values are 0.00.

CVD, cardiovascular disease; ER, emergency room; HCRU, healthcare resource utilisation; IQR, interquartile range; T2D, type 2 diabetes.

### Fig. S1 Study design for cross-sectional (A) and retrospective cohort study (B)
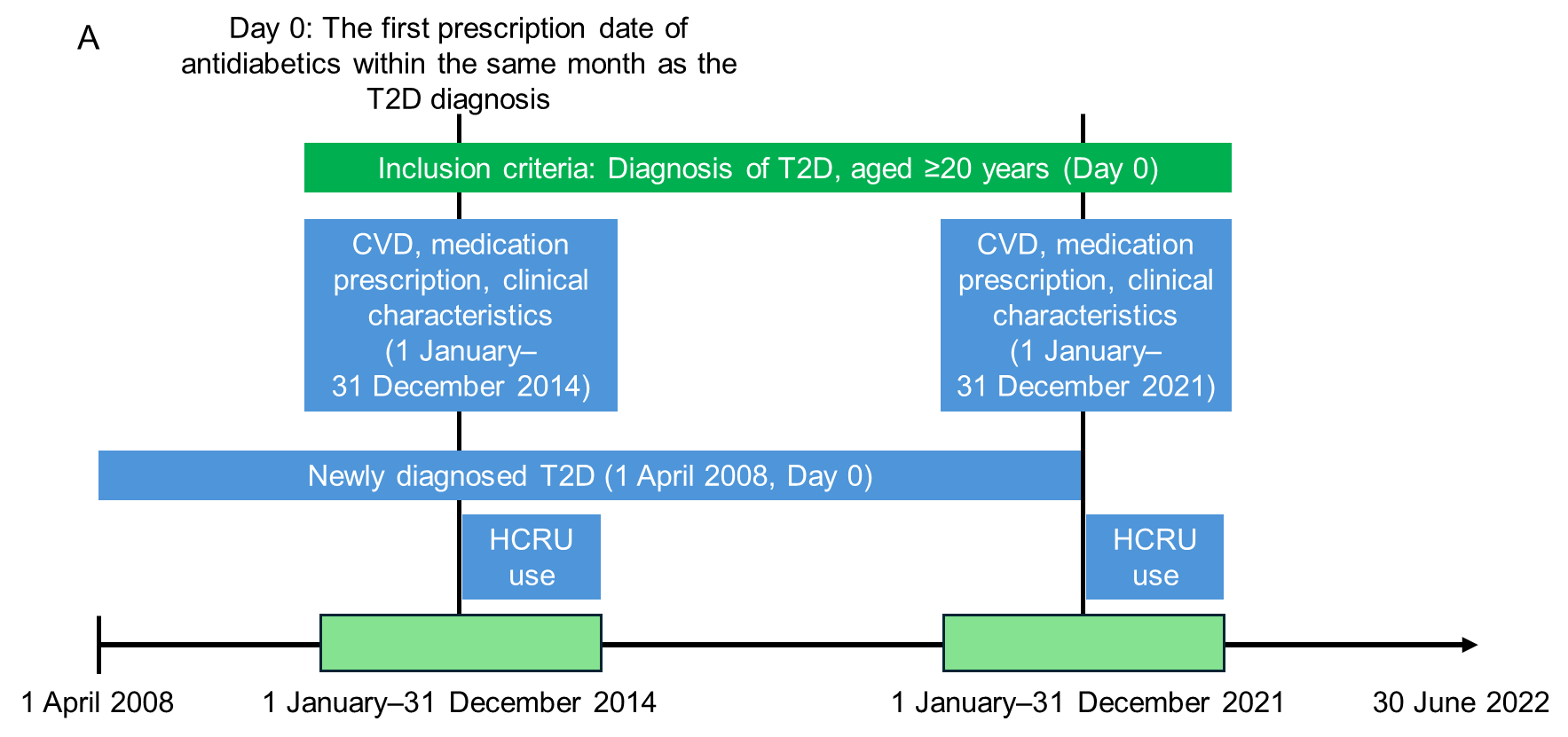

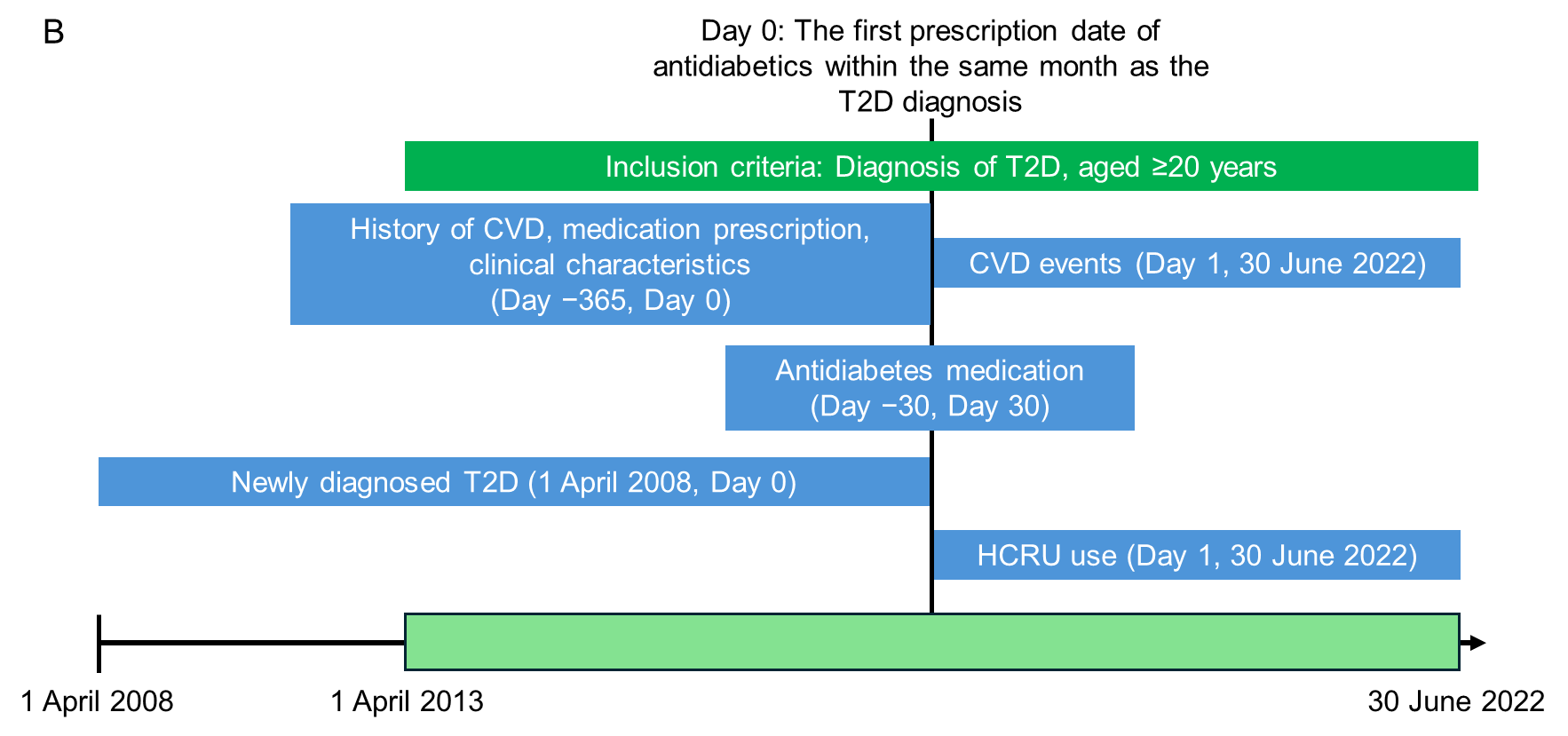


Study design of the cross-sectional study (a) and the retrospective cohort study (b). Black arrows indicate the overall study period; green boxes indicate index data windows. Data collection time windows are described within blue boxes. Newly diagnosed T2D was defined as patients without a hospital visit with a diagnostic record of T2D prior to the index date (exclusive).

CVD, cardiovascular disease; HCRU, healthcare resource utilisation; T2D, type 2 diabetes.
